# Impact of preexisting cognitive decline in patients with diabetes mellitus type II on neurocognitive outcome after cardiac surgery

**DOI:** 10.1186/1749-8090-8-S1-P154

**Published:** 2013-09-11

**Authors:** S Ivankovic, M Borojevic, I Safradin, Z Colak, I Burcar, A Hodalin, D Belina, A Lekić, H Gasparovic, B Biocina

**Affiliations:** 1 Department of Cardiac Surgery, University Hospital Rebro, Zagreb, Croatia; 2 Department of Anesthesiology and Critical Care Medicine, University Hospital Rebro, Zagreb, Croatia

## Background

Neurocognitive decline after cardiac procedure is an important and debilitating complication. The aim of our study was to evaluate the deleterious effect of diabetes mellitus type II on progression of cerebrovascular disease which may led to higler rate of neurocognitive impairment in early and late postoperative period.

## Methods

Thirty patients with diabetes mellitus type II who were scheduled for elective coronary artery bypass grafting were evaluated. As a control group, 30 patients without diabetes mellitus matched for age, EuroScore, and educational level were examined. All patients underwent a battery of neurologic and neuropsychologic tests the day before surgery, 7 days after surgery, and 4 months after surgery.

## Results

Non-diabetic and diabetic patients did not differ significantly in analysed general, clinical and perioperative characteristics, except for HbA1c values.

Preoperative neurocognitive performance, controlled for potential confounders, was lower in diabetic compared to non-diabetic patients for all tested parameters, although the difference reached statistical significance for attention domain(coefficient 12.3, 95% confidence interval 2.1-22.6, P=0.020) and motor skills values (coefficient 14.4, 95% CI 0.31-28.5, P=0.045).

One week after the surgery, the results of all evaluated neurocognitive tests significantly worsened in both patient groups. The trend was quite similar for diabetics and non-diabetics. However, neurocognitive damage was more pronounced in diabetic group. Significantly different from preoperative values obtained by linear mixed effect regression model, separately for each group (P<0.05). Four months after the surgery the values recovered in both groups, although in diabetic patients short-term, long-term memory and motor skills values did not reach preoperative values.

## Conclusion

The result demonstrated that pre-existing cognitive impairment in patients with diabetes progress and could have a significant impact on daily life functions.

